# UNDERSTANDING BARRIERS TO PALLIATIVE CARE USE AMONG ASIAN AMERICANS: A SYSTEMATIC REVIEW

**DOI:** 10.1093/geroni/igz038.3542

**Published:** 2019-11-08

**Authors:** Min Kyoung Rhee, Yuri Jang, Mona Liu, William E Haley, Susan Enguidanos

**Affiliations:** 1 University of Southern California, Los Angeles, California, United States; 2 University of South Florida, Tampa, Florida, United States

## Abstract

Despite the well-documented clinical and public health impacts of palliative care on older patients’ end of life experiences, racial and ethnic disparities in palliative care use still remain. However, empirical evidence on the sources of disparities is lacking, particularly for Asian Americans. Given the rapid growth and increasing cultural diversity of the older Asian population in the United States, a systematic review was conducted to provide a comprehensive overview of the best available evidence on the barriers to palliative care use among Asian Americans. Search was conducted using six electronic databases (PubMed, CINAHL, PsycINFO, EMBASE, Cochrane Library, and Google Scholar), and all procedures of the study have been guided by Preferred Reporting Items for Systematic Reviews and Meta-Analysis Protocols (PRISMA-P). A total of 23 studies published in the U.S. since 1998 were included. Individual level barriers to palliative care service use among Asian Americans identified by the study included lack of palliative care knowledge, lack of insurance, cultural beliefs, language barrier, attitude toward death, family centered decision-making. Provider level barriers included lack of training and experience about palliative care, patient-provider discordance in language and culture, and absence of policy or guidance for providers. Although racial disparities in palliative care for Asian Americans were evident, Asian Americans were underrepresented in palliative research. Future research effort should be directed toward the development of culturally sensitive palliative care services and policies that reduce racial and ethnic specific barriers.

